# Habitat suitability modeling to improve conservation strategy of two highly-grazed endemic plant species in saint Catherine Protectorate, Egypt

**DOI:** 10.1186/s12870-025-06401-4

**Published:** 2025-04-16

**Authors:** Mohamed M. El-Khalafy, Eman T. El-Kenany, Alshymaa Z. Al-Mokadem, Salma K. Shaltout, Ahmed R. Mahmoud

**Affiliations:** 1 Botany and Microbiology Department, Faculty of Science, Kafrelsheikh University, Kafrelsheikh, Egypt; 2 Department of Botany & Microbiology, Faculty of Science, Alexandria University, Alexandria, Egypt; 3 Oral Biology Department, Faculty of Dentistry, Pharos University, Alexandria, Egypt; 4 Chemistry Department, College of Science, Jouf University, Sakaka, Saudi Arabia; 5 Botany Department, Faculty of Women for Arts, Science and Education, Ain Shams University, Cairo, Egypt; 6 Botany and Microbiology Department, Faculty of Science, Tanta University, Tanta, 31527 Egypt; 7 Botany and Microbiology Department, Faculty of Science, Helwan University, Helwan, Egypt

**Keywords:** Climate change, Endemism, Ensemble model, Habitat suitability, Over-grazing, Saint Katherine protectorate

## Abstract

**Background:**

Biodiversity is seriously threatened by climate change impacts in the long term. Conservationists must possess a comprehensive knowledge about habitat suitability of different species and factors that control their distribution in order to effectively minimize biodiversity loss.

**Results:**

The present study showed the response of two endemic taxa in Saint Catherine protectorate (SKP) (*Micromeria serbaliana* and *Bufonia multiceps*) to anticipate climate change over the next few decades using species distribution models. In our analysis, we included the incorporation of bioclimatic variables into the SDM modeling process using four main algorithms: generalized linear model (GLM), Random Forest (RF), Boosted Regression Trees (BRT), and Support Vector Machines (SVM) in an ensemble model. The RF model outperformed other models when analyzing *Micromeria serbaliana*, whereas BRT demonstrated superiority in the case of *Bufonia multiceps*. The ensemble models exhibited the best performance, achieving a mean TSS of 0.94 for *Micromeria serbaliana* and 0.86 for *Bufonia multiceps*. *Micromeria serbaliana* was mainly affected by Mean temperature of wettest quarter (Bio8), elevation, and Aridity index. On the other hand, the most significant factors influencing *Bufonia multiceps* were determined to be Isothermality (Bio2/Bio7) × 100 (Bio3), and elevation. The habitat suitability of *Micromeria serbaliana* was slightly expanded during the period form 2041–2060, then declined again from 2061 to 2080, while it showed moderate expansion in the case *Bufonia multiceps* under the two periods.

**Conclusion:**

The results of our research support the urgent need for conservation efforts, including reintroduction and planning for in situ and ex situ conservation in appropriate habitats.

**Clinical trial number:**

Not applicable.

**Supplementary Information:**

The online version contains supplementary material available at 10.1186/s12870-025-06401-4.

## Introduction

Lack of information regarding the present range of endemic species, conservation initiatives, population characteristics, habitat and environmental conditions, risks, and other relevant information significantly impedes the effectiveness of thorough conservation initiatives designed to mitigate species extinction [1]. Some species might go extinct before we can collect precise data on them [2, 3].

Endemic species are confined to specific geographic regions due to factors such as isolation or in response to abiotic environments. Understanding endemicity is essential for establishing conservation priorities [4]. The limited geographical range of endemic taxa typically indicates higher vulnerability than other taxa, and is therefore used as a proxy for identifying conservation priorities [5]. In Egypt, there are 41 endemic species, which belong to 36 different genera and 20 families [6]. Among them, 31.7% (13 taxa) are found in Saint Catherine Protectorate (SKP). The distribution of taxa in many countries is not well understood [7, 8], primarily due to biased species collection, inadequate sampling techniques, limited research resources and facilities, and challenges in species identification and definition [9].

*Micromeria* is a genus of the Lamiaceae family, Mentheae tribe, and Nepetoideae subfamily. The leaves of Micromeria have been reported to have anti-inflammatory and antimicrobial properties [10–12]; for use in popular medicines against heart disease, headache, skin wound, infections, colds, as an antispasmodic, and as a stimulant. In Egypt, *Micromeria* is represented by five species, namely *M. serbaliana* Danin & Hedge, *M. sinaica* Benth., *M. imbricata* (Forssk.) C.Chr., *M. nervosa* (Desf.) Benth., and *M. myrtifolia* Boiss. & Hohen [13, 14]. *Micromeria serbaliana* (*Satureja serbaliana* (Danin & Hedge) Greuter & Burdet) is a narrow endemic and endangered species [15]. It was collected for first time (type specimen) by Avinoam Danin on 6 August 1968, and then it was re-recorded in 1998 by Moustafa et al. [16]. Bräuchler et al. [17]. stated that *M. serbaliana* is located in “Egypt, South Sinai: Gebel Serbal, cliffs of smooth red granite, NW exposure, 1,850 m a.s.l.”.

Various types of land use development and human activities within protected areas are identified as primary drivers of change, significantly impacting habitats, biodiversity, and the diversity and abundance of species [18]. Human activities, invasive species, and ecological factors are causing rapid transformations in rangeland ecosystems. To maintain the diversity and long-term sustainability of plant species habitats, there is an urgent requirement for a dependable prediction model capable of accurately forecasting and mapping species distribution across different ecological scenarios [19]. *Micromeria serbaliana* faces severe threats from both natural factors, such as the area’s aridity, climate change, and human activities, including the construction of dams and unmanaged building projects. A total of 20 dead and desiccated *M. serbaliana* individuals have been documented because of drought. These threats are pushing the species toward extinction [20–25]. Musa Mountain in the SCPA only had a single record of *Micromeria serbaliana* by [16]. Additionally, it faces considerable grazing pressure, which may be attributed to feral donkeys or domestic animals owned by Bedouins living near the high mountain area [26]. The SCPA is home to endemic plant species that have been significantly impacted by human activities. These activities include excessive harvesting for medicinal use or fuel, as well as overgrazing by goats, camels, and feral donkeys. Furthermore, unregulated scientific research has contributed to the problem through destructive collection practices, especially the seeds [27–29].

*Bufonia multiceps* is a plant species endemic to the Saint Katherine Protectorate (SKP), and it thrives within a specific altitude range of 1,350 to 2,624 m above sea level. Endangered status is warranted due to its limited distribution to a small region. The distribution of this species is influenced by climate change, particularly by flooding and prolonged drought. The key locations for this species within the Saint Katherine Protectorate are Wadi Gebal and Saint Catherine Mountain [30]. It is economically significant as a grazing resource for livestock [29, 31], and is used ethno-veterinary to treat digestive problems in sheep, goats, and horses [32]. *B. multiceps* faces stress from overgrazing because it is highly palatable to domestic animals [33]. Habitat quality for this species is deteriorating, leading to declines in both subpopulation sizes and the number of mature individuals. The population is fragmented, as the mountainous terrain separates the small subpopulations, many of which are poorly viable due to severe overgrazing that result in the loss of reproductive structures [34]. Khafagi et al. [29] and Omar et al. [35] identified *Bufonia multiceps* as one of the most impacted species by grazing within the SKP. It’s recorded by Omar [36] that more than 85% of the total population is affected by heavy grazing which cause a great deterioration in population distribution as well as affecting the species’ vitality. The vegetation in the species’ habitat has been disturbed by human activities, including overgrazing and uprooting [28, 29]. Observations by SKP rangers since 2000 indicate that climate change negatively impacts this species, particularly through the destructive effects of sudden flooding and prolonged drought, which have altered the species’ size, cover, sensitivity, vitality, and distribution. However, focused studies on this issue are currently lacking [28, 29]. It has been found that *B. multiceps* has an ethnoveterinary use; the whole plant is used for treatment of digestive problems [29, 35]. *Bufonia multiceps* qualifies as Endangered because it is endemic to a tiny area (with an EOO of 337 km²and an AOO of 120 km²) of the high mountain area of the St. Katherine Protectorate in southern Sinai, Egypt [34, 36].

Omar and Elgamal [1] identified the suitable habitat by Maxent modeling technique for *Micromeria serbalina* under the current environmental conditions only. They used 19 bioclimatic and 3 topographic parameters. They reported that the most suitable habitat for *M. serbaliana* was predicted to be in the middle northern and northeastern parts of the SCPA, with the highest suitability in the High Mountains and Serbal areas. The most participating variables were precipitation of driest quarter (Bio17), mean temperature of driest quarter (Bio9), and elevation. In addition, they recorded that its potential distribution based on habitat suitability was 466.1 km^2^.

The change in climate of the Sinai Peninsula exhibits a continued raise in air temperature, as well as projected changes in rainfall pattern [37]. If the combination of climate change and other human-induced changes (e.g., land use, pollution, and resources overexploitation) continues, the resilience of various ecosystems will be surpassed [38], altering their structure and function [39]. Climate change might affect the future distribution of many endemic plant species due to changes in temperature and precipitation regimes [40]. The most serious aspects of these changes are the endangerment of the occurrence of many communities and species, which may negatively affect the provision of ecosystem services. More studies are needed to understand how future climatic disturbances might influence the distribution of plant species in arid ecosystems [41].

The use of species distribution modeling (SDM) has become increasingly popular in ecological research due to its ability to predict species distributions based on their relationships with environmental factors [42]. The concept of species distribution models (SDMs) is rooted in combining environmental factors like temperature, precipitation, and land cover with data on where species are found [43–45]. SDMs use statistical and machine learning techniques to project predictions about where species are likely will to be found [46]. Maxent, Random Forest, and Boosted Regression Trees are some common algorithms used, each with unique strengths depending on the species and available data [47, 48]. Species Distribution Modeling (SDM) is a powerful tool for predicting species habitats and potential ranges, but it comes with several limitations and challenges: Limitations to the use of SDM have been suggested, such as when the data available are insufficient to inform the models as to true species distributions [49, 50], or when predictions based on extrapolations may not be robust [43, 51].

Extreme weather conditions, including drought and heat waves that are probably brought on by climate change, frequently have a profound impact on plant species and ecosystems. Species’ ecological tolerance and attributes are likely to justify different biological responses to these environmental changes. Recently, using either single modelling algorithm or ensemble of models in environmental and ecological sciences, including GLMs and GAMs (generalized linear and additive models), and machine learning techniques such as MaxEnt (maximum entropy), RF (random forest), and BRT (boosted regression tree) are now more prominent (e.g [52–54]). MaxEnt and Ensemble models are among the most used SDMs approaches in the prediction of climate change’s impact on species distribution.

Ensemble modelling is a technique that allows the use of several different modelling algorithms and combines the outcomes of all algorithms to create only one final prediction. The average model prediction is produced by weighing all the used algorithm’s predictions by an evaluation metric. Ensemble modelling is widely used for prediction of species distribution across time and space and many studies confirmed that ensemble models are preferred for the prediction processes compared to single models [55]. The use of ensemble modelling techniques was preferred over the use of the outcomes from a single modelling approach to evaluate the impact of climate changes on the range shift of species. The ensemble modelling techniques provide more robust and accurate results and avoid overfitting of the model [56]. Besides, they minimize the prediction generalization errors and reduce overfitting when modelling rare species. Ensemble modelling is considered a better alternative to single models for future climate projection modelling with large numbers of species [57].

The outcomes obtained from species distribution models are crucial for guiding conservation planning methods and making management choices. With the increasing challenges that species are experiencing due to climate change, comprehending the possible changes in their geographical range helps conservationists in identifying vulnerable areas and taking proactive actions. This could include establishing new protected zones, assisting in moving species to new locations, or carrying out habitat restoration projects based on the predicted future suitable habitats. Understanding the adaptive strategies and potential changes in distribution is essential for well-informed conservation and management approaches [58].

The biodiversity of Egypt’s plant life is at risk due to anthropogenic pressures such as overexploitation and habitat destruction. Anthropogenic activity is one of the major threats in the areas supporting the occurrence of endemic species in Sinai Peninsula due to logging, overgrazing and the rise in tourism. Cord et al. [59] compared the suitability of an existing land cover classification and spectral indices for modelling the distribution patterns of 30 Mexican trees. According to their findings, land cover based SDMs were hampered by bolder predictions and a general overestimation of suitability, which made remote sensing data substantially superior model predictors. Ahmed et al. [60] found that remote sensing and bioclimatic variables can be used to map and predict invasive species in arid/semi-arid areas. Halmy et al. [61] investigated the land use/land cover distribution in the northwestern coastal desert of Egypt using the Cellular Automata (CA)-Markov chain technique during 1988–2011. The study demonstrated that built-up, resorts, cropland, and quarrying areas expanded by about 150%, 250%, 200%, and 120%, respectively. This pattern was influenced by agriculture intensification, urban expansion, land degradation, and clearance of vegetation. The proposed model predicted expansion in quarries, urbanization of the landscape, and growth in residential areas for 2023. Gamal et al. [62] used GIS-based modelling to help in the conservation of two endangered plant species (*Ebenus armitagei* and *Periploca angustifolia*) at Wadi Al-Afreet, Egypt. Despite the national efforts in studying the predicted impacts of climate change on the geographical distribution of plant species and their dynamics, more efforts are still required. The number of such studies in Egypt is still considered low compared to studies conducted on close geographical regions. The outcomes of SDMs studies will enable the stakeholders and researchers to take prior actions towards the management and conservation of Egyptian plant species. Additionally, it can help in bridging these knowledge gaps related to the geographic distribution of rare and important plant species. The current study is designed to investigate the potential impacts of climate change on two endemic species in SKP using SDMs ensemble modeling techniques. The distribution of two endemic taxa in SKP, which are highly grazed, will be assessed in the current research to understand the impact of environmental changes using species distribution modeling.Species-distribution modelling and available environmental predictors (bioclimatic and soil parameters) will be used here for: 1- Determine the most contributed and controlled factors on the distribution of the studied species, 2- demonstrate and predict the potential distribution of the studied species under condition of climate, 3- assess the impacts of climate change on the future distribution using general circulation model IPSL-CM6ALR.

## Materials and methods

### Study area

**Fig. 1:**
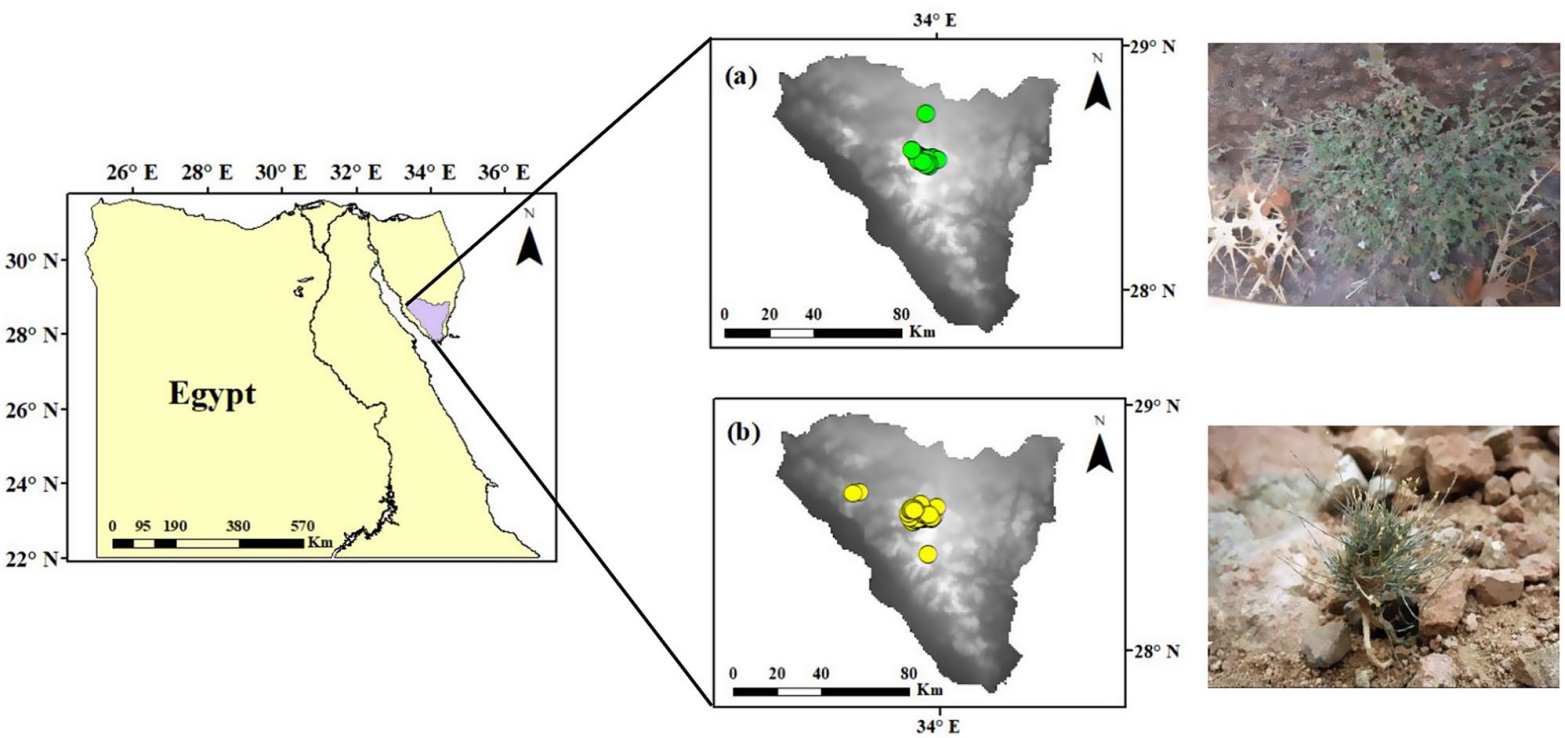
Study area surveyed for the occurrence of the studied species indicating locations of the collected occurrence records of (**a**) *Micromeria serbaliana*, (**b**) *Bufonia multiceps* collected through field surveys and other sources. The maps in Fig. 1 were produced by the authors within the framework of the GIS software package ArcGIS 10.8.2 (ESRI). The photographs were taken by the first author

The Sinai Peninsula has a distinct triangular shape. It is between the Mediterranean Sea to the north and the Red Sea to the south, and is a land bridge between Asia and Africa. Covering about 6% of Egypt’s total area, Sinai’s coastline spans approximately 700 km, distinguishing it from other Egyptian regions. Sinai encompasses almost all of Egypt’s geological formations, structures, and landforms and experiences climatic variations similar to those found elsewhere in Egypt. The mountainous terrain characterizes the southern part of Sinai, with a tableland area in the central part. In the northern region, there are two sections: the southern part has solitary dome-shaped hills and mountains, while the northern section is mainly covered by sand dunes (Fig. 1 [63], ). The distinct topography of SKP provides habitats for specific plant communities, including gorges, slopes, terraces, caves, and ridges [64, 65]. The region of SKP encounters a range of air temperatures and different amounts of rainfall. It is famous for being the most temperate area in Egypt and the sole location where snowfall occurs [65]. The average monthly temperatures range from 8.6 °C in January to 25.5 °C in August. The average annual rainfall from 1970 to 2017 was minimal and irregular, at 37.5 mm, but sudden and unpredictable flash floods with up to 300 mm of rainfall have been documented (in 2012 and 2014). The total study area cover about 10,533 km^2^.

### Field survey and occurrence data

The occurrence points of the studied species (56 points for M. serbaliana and 158 for B. multiceps) were obtained from (a) Field survey during 2016–2024; (b) the herbaria of Tanta University (TANE), Alexandria University (ALEX), Cairo University (CAI), Assiut University (ASTU), Agricultural Research Center (CAIM), Desert Research Center (CAIH), National Research Centre (CAIRC), and Kafrelsheikh University (KFSUH); (c) National Registry for Egyptian Herbaria (http://networks.asrt.sci.eg/Herbarias/Index, accessed on 10 February (2024); and (d) the Global Biodiversity Information Facility (https://www.gbif.org/, accessed on 20 March 2024). Information and data were gathered by conducting site visits to document the main habitats, coordinates, and threats. During each visit, specimens of the taxa were obtained from different locations. The identification and synonyms were established based on [13, 66, 67], and Plants of the World online (https://powo.science.kew.org/). Experimental research and field studies on plants, including the collection of plant material, comply with relevant institutional, national, and international guidelines and legislation. Identification of the plant material used in the study was performed by Prof. Dr./ Yassin M. Al-Sodany professor of plant ecology and flora at Faculty of Science- Kafrelsheikh University – Botany and Microbiology department. Some of collected samples was kept in Kafrelsheikh University Herbarium (KFSUH) (vouchers no ELKH023 to ELKH028 for M. serbaliana and ELKH008 to ELKH018 for B. multiceps).

### Bioclimatic predictors and multicollinearity

We utilized 46 bioclimatic variables downloaded from the WorldClim database (1950–2000), specifically version 2.1 [68]. The variables include 8 readings for precipitation and 11 readings for temperature, with a precision of 30 arc-seconds (approximately 1 km) [68]. The analysis involved a thorough examination of the average, minimum, and maximum values of solar radiation, precipitation, and wind speed for every month. Elevation data was sourced from the USGS National Elevation Dataset version 3.0, which was last updated in January 2022. The topographical characteristics of the research area were represented using the data (https://www.usgs.gov). Slope and aspect were derived from the elevation data using ArcGIS 10.8. Additionally, Fourteen soil variables were acquired from the Soilgrid database (https://soilgrids.org/), and the aridity index was obtained from the ENVIREM database (https://envirem.github.io/; [69, 70].

To avoid overfitting, we utilized the variance inflation factor (VIF) to identify and eliminate highly correlated variables from the SDM models. The vifcor and vifstep functions from the “usdm” package in R version 4.2.3 were used to evaluate the impact of each predictor relative to the others [71]. This approach aligns with the guidelines outlined by [72], we performed a VIF analysis and eliminated variables with VIF values exceeding five and a 0.75 correlation threshold using these functions. Furthermore, we used the getVarImp function from the “SDM” package in R version 4.3.1 to ascertain the relative importance of the predictor variables, as suggested by [73].

In order to project the range of the two species in response to climate change, we utilized the IPSL-CM6ALR global general circulation model (GCM). This model was used because it captured the observed present climate well and depicted the spatial patterns of global and zonal precipitation and temperature distribution quite well. The GCMs provide an accurate simulation of global warming and the multi-decadal variation in temperature and precipitation, predicting a higher increase in the mean annual temperature than other models over the same time interval [74, 75].This was done for the near future (2041–2060) and the far future (2061–2080) according to two different socioeconomic scenario pathways: a low scenario (SSP126) and a high scenario (585).

### Ensemble modeling and potential habitat suitability

Using the variables that resulted from VIF multicollinearity test, we utilized the occurrence data of the species using ‘usdm’ package [72], An ensemble of four modelling algorithms were chosen for constructing the ensemble species distribution model. The modelling algorithms included the generalized linear model (GLM: [76]) as parametric technique, the Boosting Regression Trees (BRT: [77]; [72]) and the random forests (RF: [78]; [79]) as non-parametric machine-learning techniques. Support Vector Machines (SVMs) are a machine learning method frequently used to build binary classifiers, also in ecological modelling ( [80]; [81]; [82]). The selected model approaches are characterized by high stability and transferability compared to other models ( [83]; [84]; [55]). Furthermore, GLM and RF behave best on both cross validation and external validation [57]. The ensemble of the species distribution models was conducted using the ‘sdm’ R package version 1.1-8 [72]. We divided the data into 70% for training and 30% for testing for model testing and evaluation according to [84]. The MTSS is recommended as threshold rule as it minimizes both the commission (over-prediction) and omission (under-prediction) errors ( [85]; [71]). The model’s accuracy was evaluated by calculating the area under the curve (AUC) and TSS, as described in the method outlined by [71]. AUC score is the dominant tool to measure the model performance, mainly due to its independence by threshold choices ( [86]; [87]; [88]). The higher the value of AUC (closer to 1), the better the performance of the model [89]. To evaluate the resulting models, several measures of accuracy assessment were estimated including the overall accuracy, sensitivity, specificity, and true skill statistics (TSS). To estimate the overall accuracy, the predictions are contrasted to a validation dataset to derive the model’s overall accuracy as follows:

$$\:\mathbf{O}\mathbf{v}\mathbf{e}\mathbf{r}\mathbf{a}\mathbf{l}\mathbf{l}\:\mathbf{a}\mathbf{c}\mathbf{c}\mathbf{u}\mathbf{r}\mathbf{a}\mathbf{c}\mathbf{y}\:=\frac{\varvec{a}+\varvec{d}}{\varvec{n}}$$

The sensitivity represents the proportion of presences accurately predicted and is estimated as follows:

$$\:\mathbf{S}\mathbf{e}\mathbf{n}\mathbf{s}\mathbf{i}\mathbf{t}\mathbf{i}\mathbf{v}\mathbf{i}\mathbf{t}\mathbf{y}=\frac{\varvec{a}}{\varvec{a}+\varvec{c}}$$

The specificity represents the proportion of absences accurately predicted and is estimated as follows:

$$\:\mathbf{S}\mathbf{p}\mathbf{e}\mathbf{c}\mathbf{i}\mathbf{f}\mathbf{i}\mathbf{c}\mathbf{i}\mathbf{t}\mathbf{y}=\frac{\varvec{d}}{\varvec{b}+\varvec{d}\:}$$

Where ***a*** is the number of the occurrence observations that were correctly predicted by the model as such, ***b*** is the number of absence observations that were wrongly predicted by the model as presence, ***c*** is the number of occurrence observations that were wrongly predicted by the model as absence, and ***d*** is number of absence observations that were correctly predicted by the model as such.

The sensitivity and specificity are insensitive to prevalence, which is the proportion of the observed sites in which the species was recorded as present. Additionally, both can be used independently for comparing and ranking models and are also independent of [90].

The true skill statistics (TSS) is estimated as follows:

$$TSS =Sensitivity + Specificity-- 1$$

The TSS values range from − 1 to + 1, where + 1 indicates perfect agreement and values of zero or less indicate a performance no better than random.

The modelling algorithms were weighted in the EM by the True Skill Statistic (TSS) and the -Maximum training sensitivity plus specificity (MTSS) threshold using sdm package in R 4.2.1 [72]. The MTSS is recommended as threshold rule as it minimizes both the commission (over-prediction) and omission (under-prediction) errors [71, 85]. The logistic output of ensemble model was a map, indexing the environmental suitability of species with values ranging from 0 (Low suitable) to 1 (high suitability). For further analysis, the ensemble models’ results were imported into ArcGIS 10.8.2 and Three classes of potential habitats were classified as follows: low, medium and high suitable ( [91]; [92]; [93]). Changes in the predicted ecological extent of the two species between the current and future climatic scenarios in correspondence of classes were computed as follows: ensemble model of current and future habitat suitability projections were converted into binary maps (presence/absence) based on the MTSS threshold. Afterward, we applied the equation (Future prediction*2) - (current prediction) to estimate the changes. The resulting output was then visualized as loss, stable, and gain areas in ArcGIS 10.8.2 as detailed in [94].

## Results

### Model performance and evolution

Our models revealed high performance of prediction with average values of AUC (0.98 ± 0.03) for *Micromeria serbaliana* and (0.97 ± 0.02) for *Bufonia multiceps* and high mean score of TSS of *Micromeria serbaliana* (0.93 ± 0.05) and *Bufonia multiceps* (0.90 ± 0.05) and other evaluation parameters showed high values (Table 1).

**Fig. 2:**
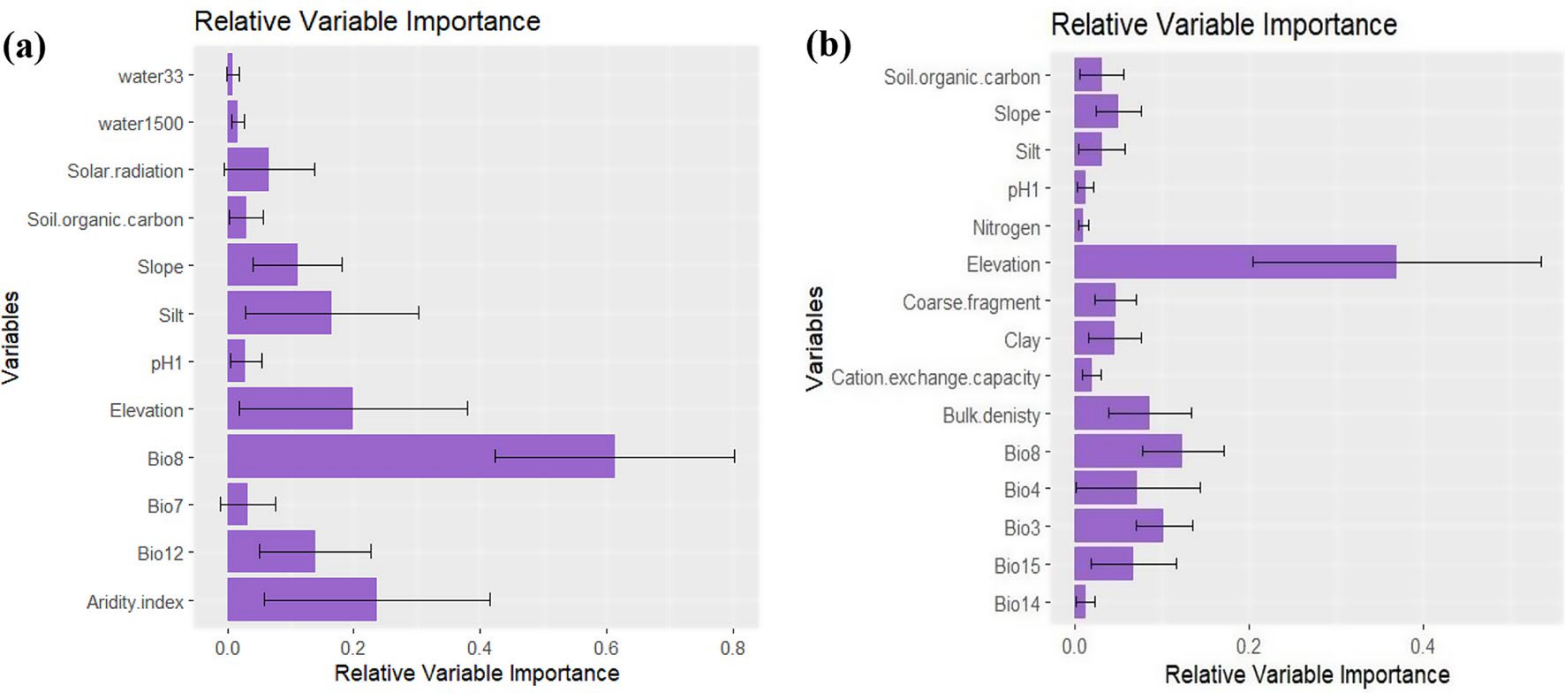
The average percentage of relative variable importance for the environmental variable used in the ensemble models predicting the potential distribution of (**a**) *Micromeria serbaliana* and (**b**) *Bufonia multiceps* under current climate conditions

Analysis of multicollinearity among the 46 predictors (supplementary Table 1) revealed that twelve variables were uncorrelated and have VIFs lower than 5 for *Micromeria serbaliana*, and fifteen variables for *Bufonia multiceps* (as shown in Table 2). These variables were utilized in the ensemble modeling process. The relative importance of the predictor variables contributing to the ensemble model showed that Bio8, aridity index, elevation, silt, Bio12 and slope were the most contributing variables that control the distribution of *Micromeria serbaliana* with contributing variable equals 61.3, 23.7, 19.9, 16.5, 13.9 and 11.2%, respectively. While *Bufonia multiceps*, the most contributing variables were elevation (36.9%), Bio8 (12.4%) and Bio3 (10.3%) (Fig. 2).

**Fig. 3:**
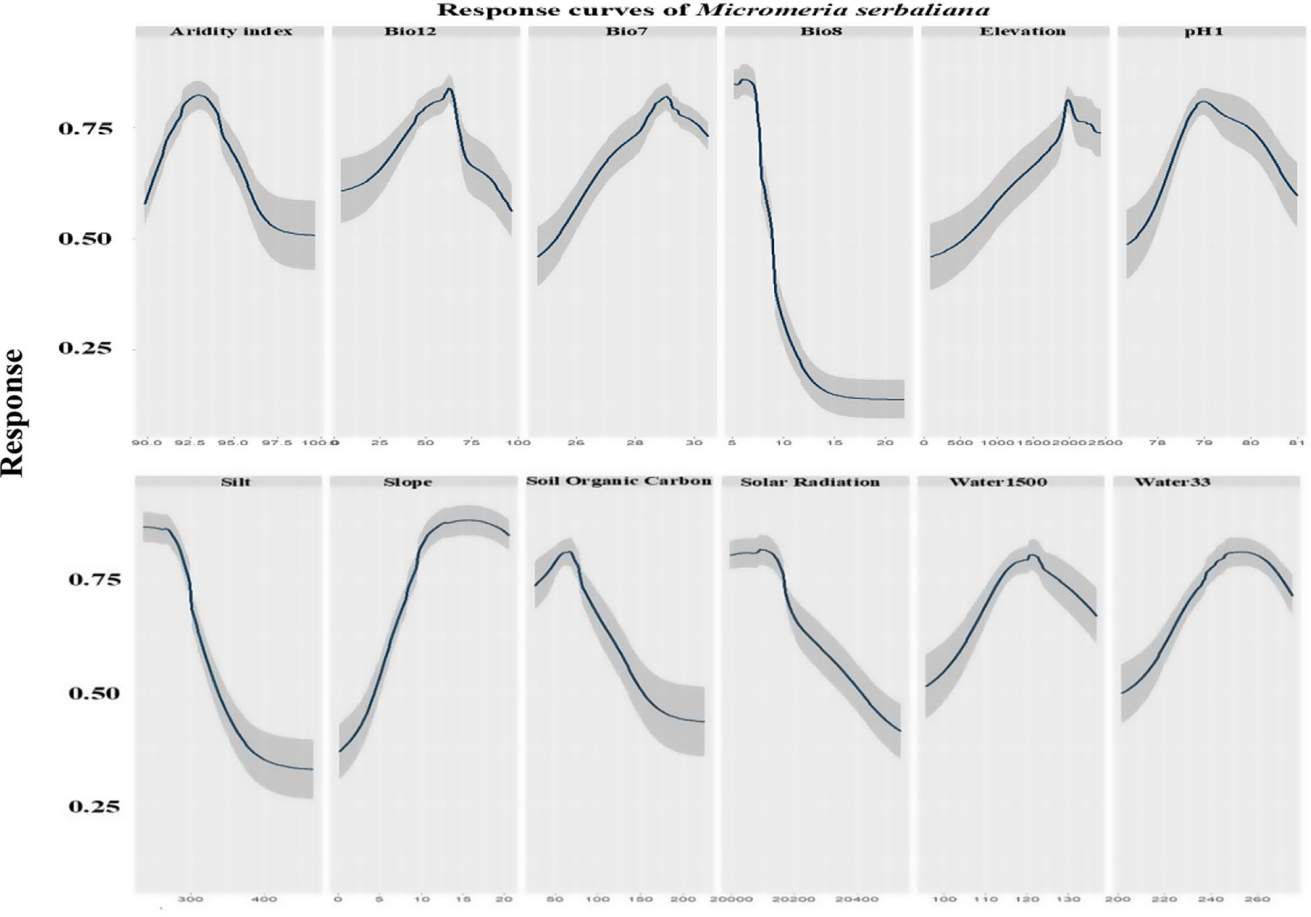
The predictor variables’ response curves were utilized in the distribution modeling of *Micromeria serbaliana*

**Fig. 4:**
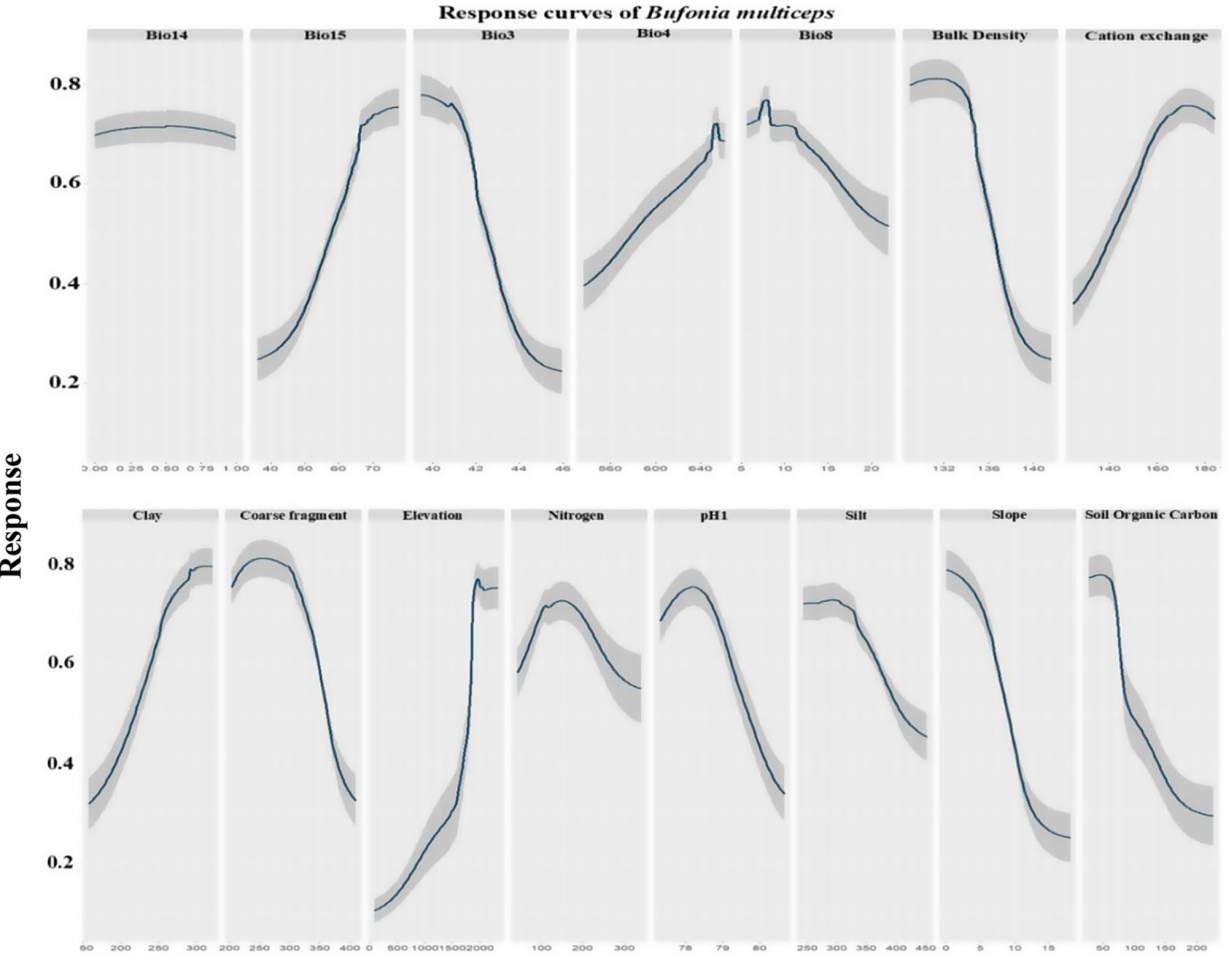
The predictor variables’ response curves were utilized in the distribution modeling of *Bufonia multiceps*

The response curves revealed the relationship between predictive variables and the logistic prediction of habitat suitability (Figs. 3 and 4). The optimum ranges of the response curves of the bioclimatic variables of *Micromeria serbaliana* showed that the aridity index range was 90 to 96, while Bio12 with peak equal 62.5 mm, Bio7 with peak equal 29 ^o^C, Bio8 with range 7.5 to 15 ^o^C, maximum elevation was 2000 m a.s.l, the range of pH was from 7.7 to 8.1, silt range was from 275 to 400 g/kg, the maximum slope range was from 10 to 20 degree, soil organic carbon range was from 25 to 70 Mg ha-1 (Fig. 3).

Regarding *Bufonia multiseps*, response curves demonstrated that an increase in Bio15 (range 30 to 70 mm), Bio4 with range 540 to 660, cation exchange capacity with range from 110 to 180 mmol(c)/kg, clay (ranged from 50 to 300 g/kg) and elevation with maximum peak was 1750 to 2000 m a.s.l led to a higher probability of presence (Fig. 4).

**Table 1:** Performance of the model algorithms for the two studied taxa

Methods	Micromeria serbaliana					Bufonia multiceps				
	BRT	GLM	RF	SVM	Average	BRT	GLM	RF	SVM	Average
**threshold**	0.24 ± 0.02	0.19 ± 0.2	0.14 ± 0.2	0.02 ± 0.01	0.15 ± 0.2	0.02 ± 0.02	0.11 ± 0.05	0.13 ± 0.1	0.06 ± 0.04	0.079 ± 0.07
**sensitivity**	0.95 ± 0.1	0.95 ± 0.04	1.00 ± 0.0	0.93 ± 0.07	0.96 ± 0.04	0.98 ± 0.03	0.96 ± 0.07	1.00 ± 0.0	0.92 ± 0.03	0.96 ± 0.04
**specificity**	0.98 ± 0.02	0.98 ± 0.01	0.94 ± 0.01	0.97 ± 0.01	0.97 ± 0.02	0.90 ± 0.02	0.95 ± 0.02	0.93 ± 0.05	0.97 ± 0.01	0.93 ± 0.03
**TSS**	0.94 ± 0.04	0.94 ± 0.05	0.94 ± 0.01	0.90 ± 0.08	0.93 ± 0.05	0.88 ± 0.05	0.91 ± 0.07	0.93 ± 0.05	0.89 ± 0.03	0.90 ± 0.05
**Kappa**	0.82 ± 0.07	0.81 ± 0.08	0.63 ± 0.1	0.68 ± 0.1	0.74 ± 0.1	0.48 ± 0.06	0.65 ± 0.08	0.63 ± 0.1	0.72 ± 0.07	0.62 ± 0.1
**Overall accuracy**	0.98 ± 0.01	0.98 ± 0.01	0.94 ± 0.04	0.96 ± 0.02	0.97 ± 0.02	0.90 ± 0.01	0.95 ± 0.02	0.94 ± 0.05	0.96 ± 0.01	0.94 ± 0.03
**AUC**	0.98 ± 0.02	0.98 ± 0.01	0.99 ± 0.01	0.95 ± 0.05	0.98 ± 0.03	0.97 ± 0.03	0.98 ± 0.01	0.99 ± 0.01	0.95 ± 0.04	0.97 ± 0.02

**Table 2:** Summary of the chosen environmental predictor variables that explain the potential distribution of *Micromeria Serbaliana* and *Bufonia multiceps* in SKP. To address multicollinearity issues, we removed correlated variables with VIF values exceeding five and a correlation threshold of 0.75

Micromeria serbaliana			Bufonia multiceps		
Code	**Variable**	**VIF**	**Code**	**Variable**	VIF
pH	pH	2.4	**Bio3**	Isothermality (Bio2/Bio7) × 100	2.01
Silt	Silt	2.8	**Bio4**	Temperature seasonality (SD × 100)	3.2
SL (%)	Slope	1.2	**Bio8**	Mean temperature of wettest quarter	3.3
Elev (m)	Elevation	2.5	**Bio14**	Precipitation of driest month	3.1
Soil organic carbon	Soil organic carbon	3.4	**Bio15**	Precipitation seasonality	2.3
Solar radiation	Solar radiation	2.6	**Bulk Density**	Bulk Density	2
water33	Vol. water content at -10kpa	1.5	**Cation exchange**	Cation exchange capacity	2.9
Water1500	Vol. water content at -1500kpa	1.6	**Clay**	Clay	2.9
Aridity	Aridity index	2	**Coarse fragment**	Coarse fragment	3.3
Bio7	Temperature annual range (Bio5-Bio6)	2.4	**Nitrogen**	Nitrogen	2.6
Bio8	Mean temperature of wettest quarter	2.6	**pH**	pH	1.2
Bio12	Annual precipitation	1.8	**SL (%)**	Slope	1.7
			**Elev (m)**	Elevation	1.9
			**Silt**	Silt	3.3
			Soil organic carbon	Soil organic carbon	4

### Current and future predictions

**Fig. 5:**
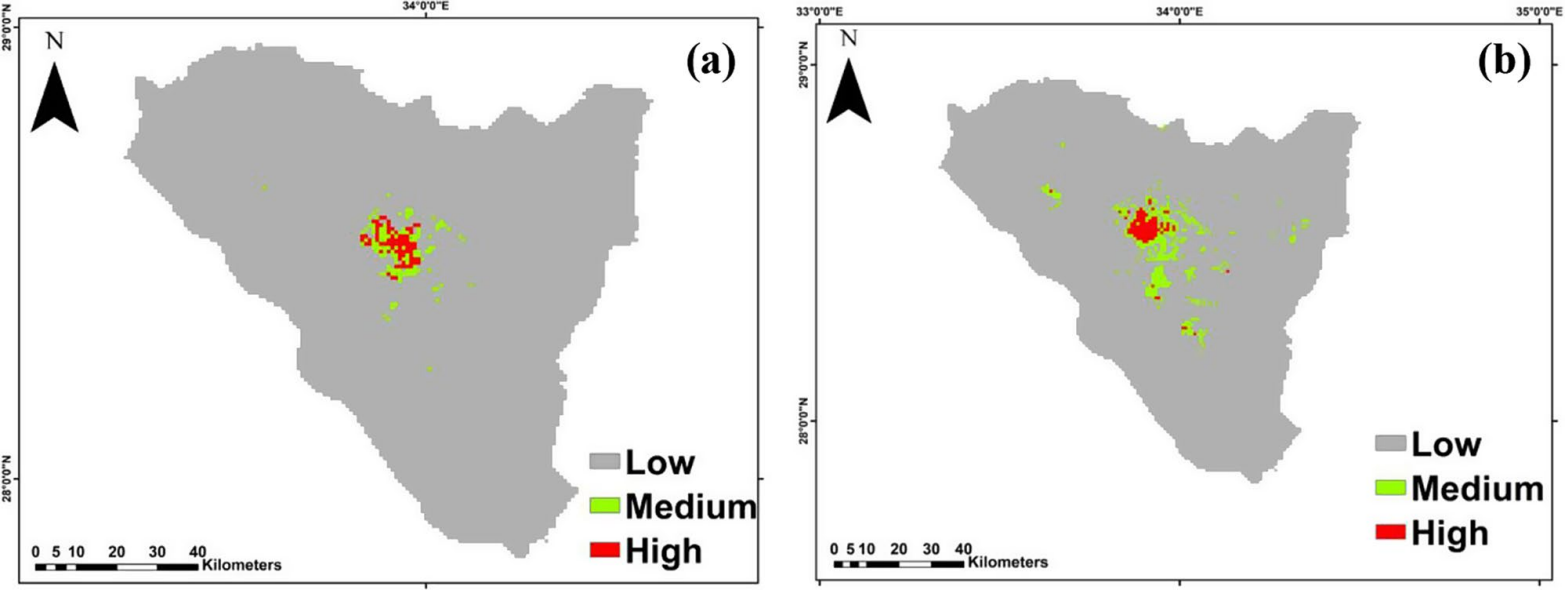
The habitat suitability map of: (**a**) *Micromeria serbaliana and (****b****) Bufonia multiceps* under the current climate conditions

The map depicting the total habitat suitability for *Micromeria serbaliana*, based on the MTSS threshold 0.076, covered an area of 194 km^2^. It indicated a high diversity of species in SKP in Abu-Mashour, Gebel Ahmar, Shag Musa, Abo Hamman and Galt Azraq (Fig. 5a; Table 3). In addition, *Bufonia multiceps* covered area of 483 km^2^, with high species diversity in Wadi Gebal, Sanit Catherine Mountain, El- Zawateen, Farsh El-Romana, Wadi El-Arbein, El-Talaa, and Al-Faraa (Fig. 5b; Table 3).

**Fig. 6:**
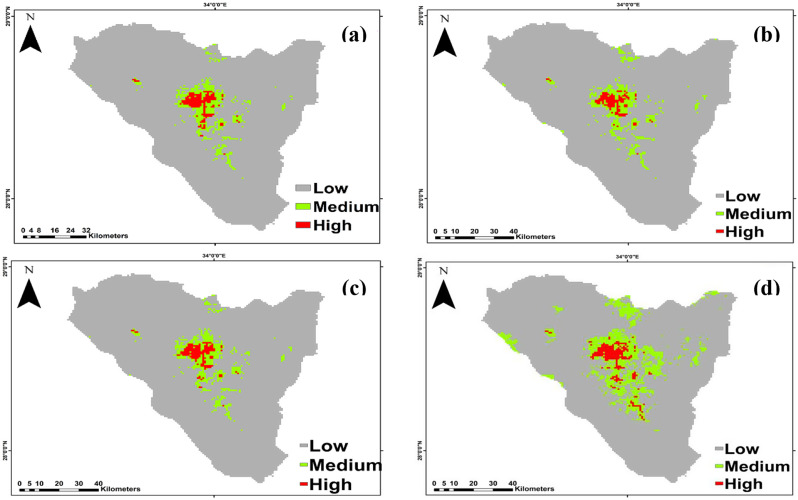
The habitat suitability map of *Micromeria serbaliana* under the two different scenarios. (**a**) SSP126 and (**b**) SSP585 projected for 2041-2060 period and (**c**) SSP126 and (**d**) SSP585 projected for 2061-2080 period

*Micromeria serbaliana* Predictions under the SSP126 scenario of the IPSL-CM6A-LR GCM model for the period 2050 and 2070 revealed a projected increase in the suitable area compared to the current distribution, with the suitable area covering 217 km^2^ and 245 km^2^ of the total study area, respectively. Otherwise, at SSP585, habitat suitability decreased with climate warming for 2050 by 180 km^2^ and 168 km^2^ for 2070 period compared to the current distribution (Fig. 6).

**Fig. 7:**
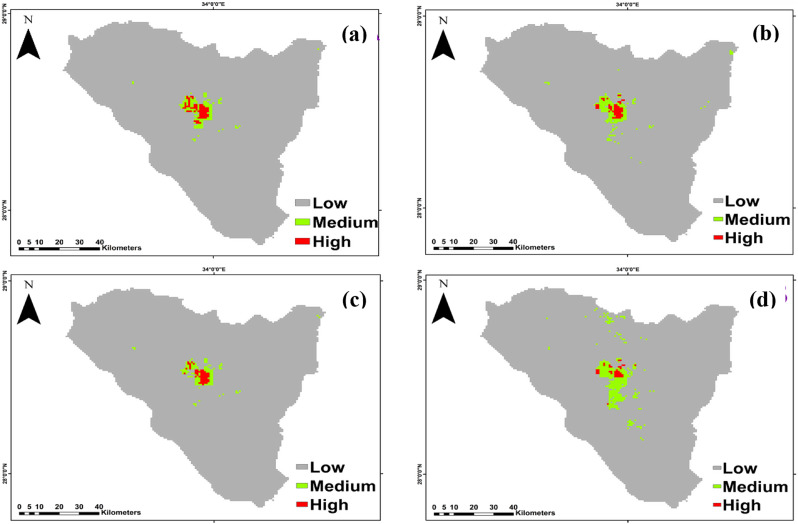
The habitat suitability map of *Bufonia multiceps* under the two different scenarios. (**a**) SSP126 and (**b**) SSP585 projected for 2041–2060 period and (**c**) SSP126 and (**d**) SSP585 projected for 2061–2080 period

The suitable area of SSP126 and SSP585 climate scenarios of the IPSL-CM6A-LR general climate model for *Bufonia multiceps* will increase under all climate scenarios, by 2050 and 2070 are shown (Fig. 7). It is predicted that the suitable habitat will increase by 654, 649, 586, and 826 km^2^ under SSP126 (2050), SSP585 (2050), SSP126 (2070) and SSP585 (2070), respectively.

**Table 3:** Comparison between current and future habitat suitability of the two studied taxa

	Micromeria serbaliana					Bufonia multiceps				
	**Habitat suitability**									
Suitability class	Current	Future				Current	Future			
		**2041–2060**		**2061–2080**			**2041–2060**		**2061–2080**	
		SSP126	SSP585	SSP126	SSP585		SSP126	SSP585	SSP126	SSP585
Unsuitable	10,339	10,316	10,353	10,288	10,365	10,050	9879	9884	9947	9707
Suitable	194	217	180	245	168	483	654	649	586	826
	**Habitat change**									
Habitat changes		Future					Future			
		**2041–2060**		**2061–2080**			**2041–2060**		**2061–2080**	
		SSP126	SSP585	SSP126	SSP585		SSP126	SSP585	SSP126	SSP585
Loss		39	60	32	62		98	99	123	76
Unsuitable		10,277	10,293	10,256	10,303		9781	9785	9824	9631
Stable		155	134	162	132		385	384	360	407
Gain		62	46	83	36		269	265	226	419

**Fig. 8:**
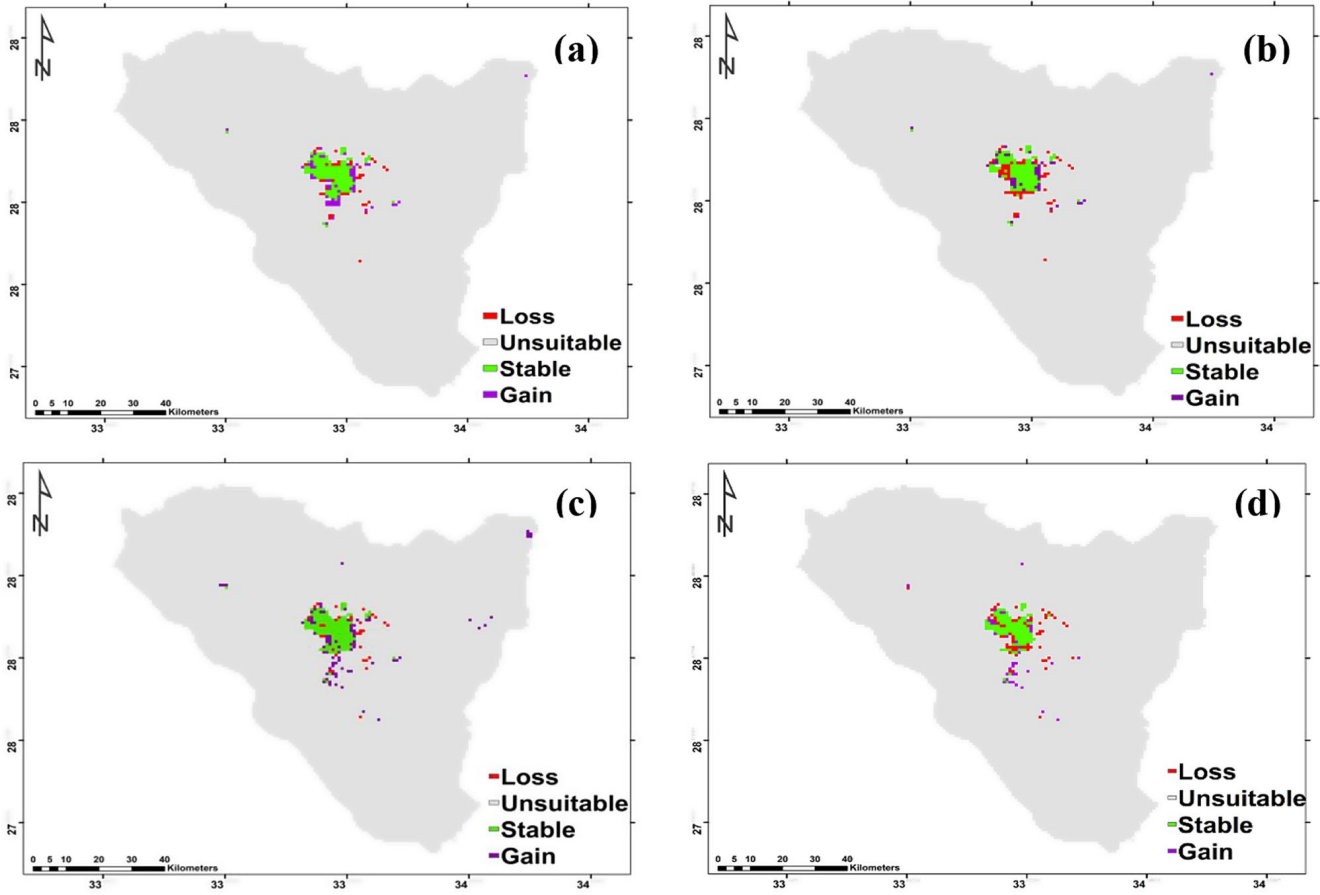
Possible habitat change under the two scenarios of climate change for *Micromeria serbaliana*. (**a**): SSP126_2041–2060, (**b**): SSP126_2061–2080, (**c**): SSP585_2041–2060, and (**d**): SSP585_2061–2080

According to two different climate change scenarios, there were differences in the potential future alterations in *Micromeria serbaliana* habitat suitability. According to both forecasts, this species’ prospective range could expand under SSP126 by 62 km^2^ and SSP585 46 km^2^ for 2050. *Micromeria serbaliana* range revealed that 39 km^2^ of the currently suitable habitats will diminish under SSP126 (the most optimistic scenario), while 60 km^2^ will be lost under SSP585 (Fig. 8a and b; Table 3). The species exhibited high richness at elevation from 2100 to 2650 m in the regions of Shag Musa, Gebel Catherine and Abo-Mashour in case of SSP126, while it exhibited high richness at elevation from 2400 to 2600 m in tiny regions of Shag Musa and Gebel Catherine in case of SSP585. By 2070 the species range will expand under SSP126 and SSP585 by 83 and 36 km^2^, respectively. The contraction of *Micromeria serbaliana* range will be 32 km^2^ under SSP126 and 62 km^2^ under SSP585. The species exhibited high richness at elevation from 2200 to 2630 m in the regions of Shag Musa and Gebel Catherine in the case of both scenarios (Fig. 8c and d; Table 3).

**Fig. 9:**
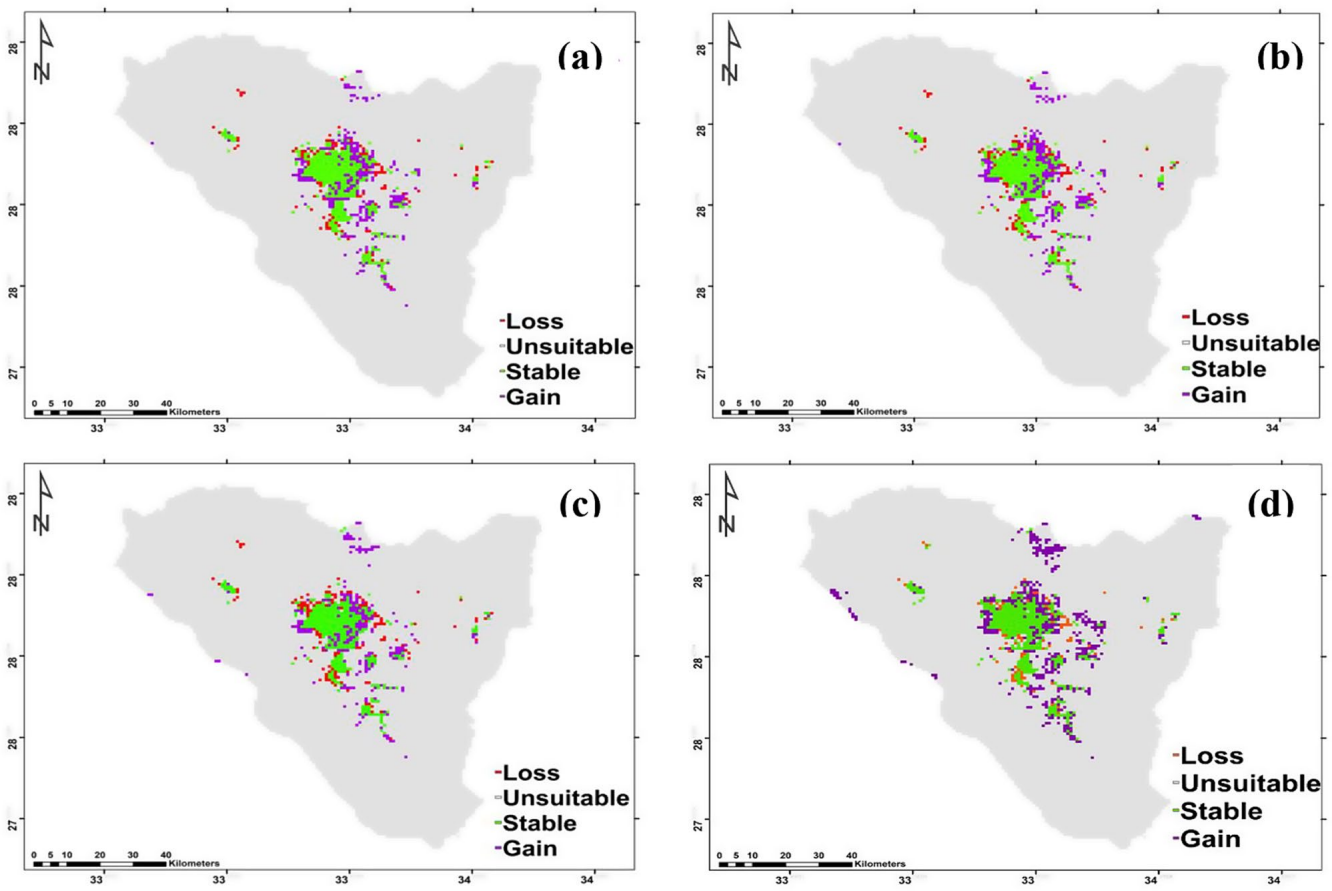
Possible habitat modifications under the two scenarios of climate change for *Bufonia multiceps*. (**a**): SSP126_2041–2060, (**b**): SSP126_2061–2080, (**c**): SSP585_2041–2060, and (**d**): SSP585_2061–2080

Based on the results of the ensemble model, the distribution pattern of *Bufonia multiceps* was projected to change under the different climate change model with different SSPs scenarios in the near and far future as compared to the current distribution pattern. The projected impact of SSP126 and SSP585 climate scenarios of the IPSL-CM6A-LR general climate model on the *Bufonia multiceps* range by 2060 and 2080 are shown. It is predicted that the species distribution range will contract by 98, 99, 123, and 76 km^2^ under SSP126 (2050), SSP585 (2050), SSP126 (2070) and SSP585 (2070), respectively. Meanwhile, it will expand by 269, 265, 226 and 419 km^2^ at SSP126 (2050), SSP585 (2050), SSP126 (2070) and SSP585 (2070), respectively (Fig. 9; Table 3). The species exhibited high richness for 2050 at Catherine Mountain region, Musa Mountain, El- Zawateen, Farsh El-Romana, Jabal Sabbah, and Jebel Serbal in both scenarios. But for 2070 it exhibited high richness at Catherine Mountain region, Musa Mountain, El- Zawateen, Farsh El-Romana, Jabal Sabbah, Jabal Ath Thabt, Jabal Umm Shawar and Jebel Serbal (Fig. 9).

## Discussion

It is essential to comprehend the spatial arrangement of biodiversity and endemism in order to plan conservation effectively [95], especially in light of the swift alteration of landscapes [96] and the influence of climate change [97]. Endemic species have restricted geographical distributions and specialized ecological niches [98]. Egypt exhibits a lower level of endemism compared to certain nations in the Middle East, similar to other dry southern countries [99].

Species distribution modeling (sdm) is a powerful tool in ecology and conservation, providing insights into the distribution of species based on environmental and spatial data. here are some key benefits: (1) conservation planning such as identifies suitable habitats (helps locate current and potential habitats for species, aiding in habitat protection), prioritizes conservation efforts (guides resource allocation by identifying areas of high conservation value) and supports endangered species protection (predicts areas where threatened species can thrive, aiding in recovery plans). (2) Understanding ecological niches by determining environmental preferences (analyzes how species interact with their environment, helping to understand their ecological requirements) and predicts species-climate relationships (identifies how environmental factors like temperature and precipitation affect species distribution), (3) assessing climate change impacts by forecasts future distributions: predicts how climate change may shift species’ ranges over time. identifies climate refugia: locates areas where species may persist despite changing climate conditions and helps in adaptation strategies; provides data for conservationists and policymakers to mitigate climate-related biodiversity loss.4) biodiversity and ecosystem management by enhances biodiversity monitoring; provides data on species richness and distribution patterns and informs land use planning; helps balance development with biodiversity conservation.

The assessment of models’ performance and the understanding of their limitations and uncertainty (Data Uncertainty: Errors or biases in input data can propagate through the model, Model Uncertainty: The choice of model architecture and assumptions affects predictions, Parameter Uncertainty: Variability in estimated parameters can impact results and Environmental or External Factors: Unpredictable external influences can introduce further uncertainties) are necessary to prevent misuse of models’ outcomes and avoid oversights in habitat prioritization for conservation and reserve design [100, 101]. Plants vary in their responses to climate changes depending mainly on their physiological or phenological characteristics [102]. The effects of climate change are being observed in the form of shift and change in species ranges [103].

The utilization of the ensemble models was also recommended over the use of the outcomes from one modelling approach to assess the effect of climate changes on the range shift of species [56, 104]. Studies have shown that an ensemble technique provides advantages over a single algorithm approach [105, 106]. The utilization of the ensemble modelling is believed to lower the uncertainty and enhance robustness, while avoiding model overfitting [56, 107, 108]. Many studies, such as [109–113] use species distribution models to determine suitable areas for plant species presence.

### Model performance, validation and variable contribution

Our study has extensively examined how climate change impacts the local extinction, colonization, and distribution of endemic plants. Accurately assessing the distribution of a species is essential in order to forecast its possible dispersal and assessing how changing ecosystems are impacted ecologically [114]. The current study utilizes species distribution models to predict potential shifts in species distributions caused by climate change. (SDMs). The outcomes of the ensemble model used in the current study were very accurate as indicated by all the measures recommended for assessing models’ performance. The Area Under the Curve (AUC) of the Receiver Operating characteristic Curve (ROC) was estimated to evaluate the accuracy of the resulting models. AUC score is one of the key measures used for assessing SDMs model performance, mainly due to its insensitivity to threshold selection [86–88]. The closer the value of AUC to 1, the better the performance of the model [89, 115]. The generated AUC graph generated by plotting the true positive predictions (sensitivity) against the false positive predictions (1-specificity) [116]. The selected environmental variables in the present study resulted in a robust model with high AUC and TSS values and other evaluation parameters, indicating a high predictive power for the relationship between environmental variables and the spatial distribution of the *Micromeria serbaliana* and *Bufonia multiceps* in Saint Catherine evidenced from an independent test dataset.

Multiple factors, such as physical, chemical, and biological aspects, determine the distribution of species [117]. Plant and animal geographic dispersion can be influenced by soil temperature, moisture content, and nutrient availability [118]. Based on the results of the current research and Abdelaal et al. [119], climatic variables indicating seasonal patterns have a more significant impact on the distribution of species at local scales. Our prediction showed that variables that have the greatest significance in explaining the possible distribution of *Micromeria serbaliana* and *Bufonia multiceps* in Saint Catherine including the Bio8, aridity index, elevation, silt, Bio12 and slope were the most contributing variables that control the distribution of *Micromeria serbaliana.* This indicates that the influence of temperature on *Micromeria serbaliana* was stronger than that of precipitation. Based on the response curves, the probability of occurrence increased with the increase in aridity index, Bio12, Bio7, elevation, pH, slope and soil water content while decreased with the increase in Bio8, silt, soil organic carbon and soil radiation. Elevation, Bio8 and Bio3 that control the distribution of *Bufonia multiceps.* These results confirmed with Omar 2017 results that the most factors affecting the distribution of *Bufonia multiceps* are temperature related variables and altitudes.

The response curves revealed that probability of presence decreased with an increase in Bio3, Bio8, Bulk density, Nitrogen, silt and slope while the probability of distribution increased by Bio15, Bio4, cation exchange, clay and elevation. The present study elucidated those climatic variables, and topographic variables may be regarded as limitation variables for the potential geographic distribution of the two plant species in Saint Cathrine. This result can be reinforced by that in mountainous areas; vegetation reacts to minute variations in the topography, such as slope, which have an impact on microclimatic conditions including soil moisture and temperature [120]. Furthermore, the variation in soil properties in Saint Cathrine could be influenced by the slopes, topography, and vegetation composition [121]. The results align with the study by Dubuis et al. [122], which utilized topo-climatic variables along with edaphic variables to predict the distribution of 115 plant species in the western Alps of Canton deVaud, Switzerland. The study found that all three types of variables have an impact on the distribution of plants. Furthermore, Lannuzel et al. [123] modeled 23 out of the 25 rarest species from Mount Kaala, a narrow-endemism hotspot in New Caledonia, to investigate potential changes in their current distribution. The distribution of the species was influenced by these variables. The arrangement of flora in elevated landscapes is significantly impacted by temperature and rainfall, as shown by the research conducted by [124]. This is agreed with Abdelaal et al. [119] who reported that Bio14, elevation, and pH were the most influential for the distribution of *Primula boveana* (endemic to SKP). In addition, Omar and Elgamal [1] found that the precipitation during the driest quarter (Bio17), the average temperature during the driest quarter (Bio9), and the elevation have the greatest impact on the distribution of *Micromeria serbaliana* in SKP.

### Current and future predictions

The study outcomes based on the current data showed that the of *Micromeria serbaliana* current suitable habitat can be found in Abu-Mashour, Gebel Ahmar, Shag Musa, Abo Hamman, and Galt Azraq. Similarly, *Bufonia multiceps* is situated in Wadi Gebal, Sanit Catherine Mountain, El-Zawateen, Farsh El-Romana, Wadi El-Arbein, El-Talaa, and Al-Faraa. Omar and Elgamal [1] reported that *Micromeria serbaliana* is distributed in two locations in SKP (high mountain and Gebel Serbal areas). Furthermore, according to Omar [36], *Bufonia multiceps* in the SKP region is primarily found in Saint Catherine Mountain and Wadi Gebal.

Habitat suitability decline due to future global warming for the year 2070 is predicted for *Micromeria serbaliana*. The species limited to mountaintops and specific ranges are expected to adjust their range limits by moving towards higher elevations in response to projected global warming [125]. As a result, the anticipated global warming could have negative impacts on native species that presently exist at the highest elevation of the St. Catherine Mountains [126].This is in agreement with Omar and Elgamal [1], who expected such a decline for *Micromeria serbaliana* in SKP. In addition, Abdelaal et al. [119] predicted that habitat suitability of *Primula boveana* will be declined due to future global warming for the years 2050 and 2070. Moreover, recent research conducted in the identical region supported the observation of changes in the geographic range for the native *Rosa arabica* plant [127]. Prolonged drought, abrupt flooding causing uprooting, and overgrazing will result in the loss of a portion of the habitat, affecting the size, cover, sensitivity, vitality, and spread of the species. Consequently, the habitat of this species will become fragmented [30]. The fragmentation could stem from the current distributions of species, particularly within the intermountain wadis and high elevation ridges. These mountainous physical barriers hinder gene flow, leading to the formation of long-lasting isolated subpopulations. This concept is also supported by Pennington et al. [128] and Särkinen et al. [129].

On the other hand, habitat suitability expansion for the years 2050 and 2070 is predicted for *Bufonia multiceps*. Most of the gained areas in our mode were located in the high-elevated regions. Climate change has affected the distribution of various species, although the effects vary depending on the species [130, 131]. Mountainous plant communities around the world have been found to be diverse. Species are moving to higher elevations to benefit from increased precipitation and cooler temperatures, leading to improved plant growth as a consequence [132, 133]. It is expected that this species’ range will expand as a result of climate change. Climate change has been observed to be causing endemic herbs in Namibia to expand their range [134], Certain endemic plants are found in biodiversity hotspots. plant species in Sardinia, Italy [135], many species of Larix [136], India [137], and a few European plant species [138].

Moreover, our findings agree with Wilson et al. [139] and Chen et al. [140] that reported that in response to climatic changes, animal and plant species have shown recent alterations in both latitudinal and altitudinal distributions, with ranges growing at high latitudes and altitudes and shrinking at lower latitudes and altitudes. *Bufonia multiceps* is expected to disappear in the high elevated regions. This is due to the high grazing and over collecting in SKP.

In addition, based on criteria that take into account uniqueness (including near-endemics, steno- and national endemics), diversity of species, and fragility of species (such as threatened species at global, regional, and national levels), SKP is recognized as one of the most significant conservation areas in Egypt [23]. The majority of Egypt’s endemic plants are encompassed within it. It is not too difficult to define the restrictions of SKP’s priority conservation regions. Conservation plans should incorporate creative community-public-private conservation collaborations for maximum preservation. The identified hotspots may be kept either individually or as a connected whole within the existing network of protected areas, especially in areas with substantial environmental fragmentation.

This study represents a significant effort to assess the geographical niche of the SKP endemic species, *Micromeria serbaliana* and *Bufonia multiceps*. The anticipated impacts of climate change must be integrated into conservation and management strategies for threatened SKP endemic species. The study highlights the significant contribution of bioclimatic predictors in determining the geographic distribution of *Micromeria serbaliana* and *Bufonia multiceps*. Future research should incorporate additional factors such as soil characteristics, anthropogenic impacts, interspecies competition, socioeconomic considerations, and topography to develop a more comprehensive understanding of the species’ habitat requirements. Overall, this study provides valuable insights into the potential changes in *Micromeria serbaliana* and *Bufonia multiceps* habitats under various climate change scenarios, emphasizing the urgent need for conservation measures to protect this species and sustain the ecosystems it supports.

## Conclusion

The study involved simulating the suitable habitats for two endemic taxa in SKP that experience heavy grazing, both currently and in the future. Additionally, the study evaluated the potential impact of climate change on the future distribution of these taxes using an ensemble model. Our findings suggest that climate change is likely to lead to variations in the suitability of habitats for these two species. It is crucial to establish comprehensive conservation strategies, with a specific emphasis on conserving and rehabilitating habitats, promoting sustainable land management practices, and addressing and adapting to climate change.

## Electronic supplementary material

Below is the link to the electronic supplementary material.

Supplementary Material 1

## Data Availability

The datasets used and/or analyzed during the current study are available from the corresponding author on reasonable request.
