# Supplementary material for: Habitat suitability modeling to improve conservation strategy of two highly-grazed endemic plant species in saint Catherine Protectorate, Egypt
Source: BMC Plant Biol. 2025 Apr 16;25:485. doi: 10.1186/s12870-025-06401-4 (PMC12001567; doi:10.1186/s12870-025-06401-4)
Supplement: Supplementary file 1 — Supplementary Material 1 [file 12870_2025_6401_MOESM1_ESM.docx]

**Table S1.** Environmental variables used in the study

| Variable | Code | Source | units |
| --- | --- | --- | --- |
| Climatic/Bioclimatic variables | | | |
| Annual mean temperature | Bio1 | WorldClim | °C |
| Mean diurnal range (max. Temp- min. temp) | Bio | WorldClim | °C |
| Isothermality (Bio2/Bio7) × 100 | Bio3 | WorldClim | °C |
| Temperature seasonality (SD × 100) | Bio4 | WorldClim | °C |
| Max temperature of warmest month | Bio5 | WorldClim | °C |
| Min temperature of coldest month | Bio6 | WorldClim | °C |
| Temperature annual range (Bio5-Bio6) | Bio7 | WorldClim | °C |
| Mean temperature of wettest quarter | Bio8 | WorldClim | °C |
| Mean temperature of driest quarter | Bio9 | WorldClim | °C |
| Mean temperature of warmest quarter | Bio10 | WorldClim | °C |
| Mean temperature of coldest quarter | Bio11 | WorldClim | °C |
| Annual precipitation | Bio12 | WorldClim | mm |
| Precipitation of wettest month | Bio13 | WorldClim | mm |
| Precipitation of driest month | Bio14 | WorldClim | mm |
| Precipitation seasonality | Bio15 | WorldClim | mm |
| Precipitation of wettest quarter | Bio16 | WorldClim | mm |
| Precipitation of driest quarter | Bio17 | WorldClim | mm |
| Precipitation of warmest quarter | Bio18 | WorldClim | mm |
| Precipitation of coldest quarter | Bio19 | WorldClim | mm |
| minimum temperature | tmin | WorldClim | °C |
| maximum temperature | tmax | WorldClim | °C |
| average temperature | tavg | WorldClim | °C |
| Precipitation | Prec | WorldClim | mm |
| wind speed | Wind | WorldClim | m s^-1^ |
| water vapor pressure | Vapr | WorldClim | kPa |
| Climatic moisture content |  | ENVIREM | - |
| Solar radiation | Srad |  | kJ m^-2^ day^-1^ |
| Degree of water deficit below water need | Aridity index | ENVIREM | - |

**Table S1. (Continued)**

| Variable | Code | Source | units |
| --- | --- | --- | --- |
| Topographic variables | | | |
| Elevation | Elev (m) | WorldClim | m |
| Slope | SL (%) | derived from elevation | (%) |
| Aspect | AS (degrees) | derived from elevation | degree |
| Soil factors | | | |
| Organic carbon density |  | Soilgrid | g/dm^3^ |
| Soil organic carbon stock |  | Soilgrid | g/m² |
| Bulk Density |  | Soilgrid | cg/cm^3^ |
| Caly content |  | Soilgrid | g/kg |
| Coarse fragment |  | Soilgrid | cm^3^/dm^3^ |
| Sand |  | Soilgrid | g/kg |
| Silt |  | Soilgrid | g/kg |
| Cation exchange capacity |  | Soilgrid | mmol(c)/kg |
| Nitrogen |  | Soilgrid | cg/kg |
| Soil organic carbon |  | Soilgrid | g/kg |
| pH water |  | Soilgrid | pH × 10 |
| Vol. water content at -10kpa | Water10 | Soilgrid | m³/m³ |
| Vol. water content at -33kpa | Water33 | Soilgrid | m³/m³ |
| Vol. water content at -1500kpa | Water1500 | Soilgrid | m³/m³ |
